# Assessment of Geriatric Problems and Risk Factors for Delirium in Surgical Medicine: Protocol for Multidisciplinary Prospective Clinical Study

**DOI:** 10.2196/59203

**Published:** 2025-01-22

**Authors:** Henriette Louise Möllmann, Eman Alhammadi, Soufian Boulghoudan, Julian Kuhlmann, Anica Mevissen, Philipp Olbrich, Louisa Rahm, Helmut Frohnhofen

**Affiliations:** 1 Department of Oral-, Maxillo- and Plastic Facial Surgery Heinrich-Heine-University Duesseldorf Düsseldorf Germany; 2 Heinrich-Heine-Universität Düsseldorf Universitätsstrasse 1 Düsseldorf Germany; 3 Orthopedics and Trauma Surgery University Hospital Düsseldorf Düsseldorf Germany

**Keywords:** delirium, older patients, perioperative assessment, age-related surgical risk factors, geriatric assessment, gerontology, aging, surgical medicine, surgical care, surgery, multidisciplinary, prospective study, perioperative, screening, palliative care, health informatics

## Abstract

**Background:**

An aging population in combination with more gentle and less stressful surgical procedures leads to an increased number of operations on older patients. This collectively raises novel challenges due to higher age heavily impacting treatment. A major problem, emerging in up to 50% of cases, is perioperative delirium. It is thus vital to understand whether and which existing geriatric assessments are capable of reliably identifying risk factors, how high the incidence of delirium is, and whether the resulting management of these risk factors might lead to a reduced incidence of delirium.

**Objective:**

This study aimed to determine the frequency and severity of geriatric medical problems in elective patients of the Clinics of Oral and Maxillofacial Surgery, Vascular Surgery, and Orthopedics, General Surgery, and Trauma Surgery, revealing associations with the incidence of perioperative delirium regarding potential risk factors, and recording the long-term effects of geriatric problems and any perioperative delirium that might have developed later the patient’s life.

**Methods:**

We performed both pre- and postoperative assessments in patients of 4 different surgical departments who are older than 70 years. Patient-validated screening instruments will be used to identify risk factors. A geriatric assessment with the content of basal and instrumental activities of daily living (basal activities of daily living [Katz index], instrumental activities of daily living [Lawton and Brody score], cognition [6-item screener and clock drawing test], mobility [de Morton Mobility Index and Sit-to-Stand test], sleep [Pittsburgh Sleep Quality Index and Insomnia Severity Index/STOP-BANG], drug therapy [polypharmacy and quality of medication, Fit For The Aged classification, and anticholinergic burden score], and pain assessment and delirium risk (Delirium Risk Assessment Tool) will be performed. Any medical problems detected will be treated according to current standards, and no intervention is planned as part of the study. In addition, a telephone follow-up will be performed 3, 6, and 12 months after discharge.

**Results:**

Recruitment started in August 2022, with 421 patients already recruited at the time of submission. Initial analyses of the data are to be published at the end of 2024 or the beginning of 2025.

**Conclusions:**

In the current study, we investigate whether the risk factors addressed in the assessment are associated with an increase in the delirium rate. The aim is then to reduce this comprehensive assessment to the central aspects to be able to conduct targeted and efficient risk screening.

**Trial Registration:**

German Clinical Trials Registry DRKS00028614; https://www.drks.de/search/de/trial/DRKS00028614

**International Registered Report Identifier (IRRID):**

DERR1-10.2196/59203

## Introduction

### Background

From the advances in surgical medicine with the development of less stressful surgical and anesthetic procedures, more and more older people are receiving surgical care who would not have been treated surgically a decade ago [1]. However, older age is associated with numerous additional problems, such as frailty [2], cognitive impairment [3,4], sleep disorders [5,6], and polypharmacy [7,8]. These age-associated problems are not routinely recorded but make older patients vulnerable. Perioperative delirium of vulnerable older patients is a major problem. Delirium is distressing for the individual, increases the burden of care, and has long-term negative consequences in terms of cognition, self-care ability, and prognosis [9].

Thus, incidences of perioperative delirium in older people are reported to be as high as 50% [10,11]. Factors influencing the incidence of delirium include duration and depth of anesthesia, the size of the surgical procedure, the duration of the preoperative fasting period, preoperative blood pressure, and frailty, brain disorders, hearing impairment, or history of sleep disturbances [12].

Perioperative delirium is a relatively common and serious complication after surgery [13-15]. The *DSM-5* (*Diagnostic and Statistical Manual of Mental Disorders* [Fifth Edition]) includes delirium into the broader category of neurocognitive disorders, which include acquired cognitive dysfunction [16,17]. Delirium is defined as an attention deficit disorder that develops over a short period of time, with additional cognitive impairment that cannot be explained by other preexisting neurocognitive impairments [18]. According to the *DSM-5*, the typical symptoms that characterize delirium are (1) “disturbance in attention,” (2) “disturbance develops over a short period of time,” (3) “additional disturbance in cognition,” (4) “disturbances in the first 2 criteria are not better explained by another preexisting disorder,” and (5) “disturbance is a direct physiological consequence of another medical condition, substance intoxication, or withdrawal” [19,20]. In general, the incidence of delirium in surgical procedures is 2%-3%, and in high-risk patients, it is even much higher at 50%-70% [11,21]. Nevertheless, precise causes as well as risk factors and incidences for the development of postoperative delirium are not yet known or deciphered [10].

In the literature, several potential risk factors for postoperative delirium are indicated. Among these are the duration and depth of anesthesia [22,23], the extent of the surgical procedure, the duration of the preoperative fasting period [10,24], the preoperative blood pressure, as well as frailty, brain disorders, hearing disorders, or sleep disorders in the history [2,25]. Delirium with its multifactorial genesis is a major challenge in risk stratification and diagnosis. Validated screening tools exist for many of the risk factors [26-32]. However, recording all risk factors is not effective in everyday clinical practice and is difficult to implement in terms of time. It is therefore necessary to compile a specialist assessment in order to identify patients at risk of delirium as effectively and reliably as possible.

Some of these risk factors are captured by validated screening instruments. Identifying risk factors allows better preoperative management [10]. Still, the regular recording of dementia, the need for assistance in daily life, or the accumulation of several diseases by geriatric screening to determine geriatric care needs is practically not established. Comorbid geriatric problems leading to functional limitations are therefore often overlooked, although they represent a major social and economic problem [33,34].

### Aim of This Research

We aim to analyze which parameters of our comprehensive geriatric assessment are associated with the occurrence of delirium depending on the specialty, and what parameters are to be included in a time-effective, target-oriented, and implementable assessment.

## Methods

### Study Design and Setting

This prospective clinical observational study analyzes the frequency and risk factors for perioperative delirium in patients after surgical treatment in the Department of Orthopedics and Trauma Surgery, the Department of Vascular and Endovascular Surgery, General Surgery, and the Department of Oral and Maxillofacial Plastic Surgery of the University Hospital of Düsseldorf in the intensive care unit and normal ward. The University Hospital of Düsseldorf is the largest hospital in the state capital of North Rhine-Westphalia, Germany. It houses more than 50,000 in-patients per year [35].

### Study Population

Inclusion criteria will be patients (aged 70 years and older) presenting for elective and emergency surgery at the Departments of Oral and Maxillofacial Surgery, Orthopedics and Trauma Surgery, General Surgery, and Vascular Surgery. In addition, we will also contact the included patients by telephone 3, 6, and 12 months after discharge to ask about their health status in a standardized way. No interventions are planned as part of this study, and any medical problems uncovered will be treated according to standard medical practice.

A case number calculation is hardly possible due to the sparse data. Therefore, one goal of this study is to collect robust data on the incidence of delirium and to be able to plan further studies on the basis of this solid data. Initially, we will offer participation in this study to all consecutive presenting patients who meet the inclusion criteria (Textbox 1). The initial recruitment period is planned to be 6 months. The goal is to enroll at least 100 patients per discipline, in total over 400 patients. If this number is not reached within this period, the recruitment period will have to be extended accordingly. Solid incidence figures are to be determined within the framework of this pilot study, so further studies can then be planned with possible case number calculation.

**Inclusion and exclusion criteria.**

**Inclusion criteria**

- Patients presenting for elective surgery at the Clinic for Oral and Maxillofacial Plastic Surgery, Orthopedics and Trauma Surgery, General Surgery, and Vascular Surgery.
- Age 70 years.
- Consent of the patient or caregiver.

**Exclusion criteria**

- No consent to participate.
- Unstable clinical situation.
- Patients requiring palliative care.
- Very advanced dementia (Reisberg VI-VII).

### Recruitment

**Figure 1:**
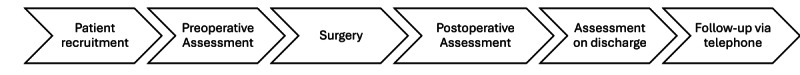
Study procedure overview. Timeline including recruitment, pre- and postoperative assessment, surgery, discharge assessment, and the assessment by phone after 3,6, and 12 months.

Patient recruitment starts in August 2022. We have been in negotiations with other surgical disciplines for almost a year, aiming to include as many different surgical specialties as possible. We are therefore submitting this study protocol during ongoing recruitment. After inpatient admission in the respective disciplines, the eligible patients are informed about the clinical examination by the study doctor. The patients are given sufficient time to think about their participation and to discuss it with their caregivers. They can also clarify any questions with the study doctor. After the patient has given and signed his or her consent, he or she takes part in the study (Figure 1).

### Measures and Parameters

In the context of this study, in addition to routine preoperative preparation in elective and emergency older patients, a geriatric assessment (Textbox 2) with the content of basal and instrumental ADL (basal ADL: Katz index [26]; instrumental ADL: Lawton and Brody score [27]; emotion: World Health Organization-5 scale [28]; cognition: 6-item screener and clock drawing test; mobility: de Morton Mobility Index and Sit-to-Stand test [29]; sleep: Pittsburgh Sleep Quality Index, Insomnia Severity Index, and STOP-BANG [30-32]; drug therapy: polypharmacy and quality of medication, Fit For The Aged classification, and anticholinergic burden score), pain assessment, and delirium risk (Delirium Risk Assessment Tool) is to be performed. The Fit For The Aged list evaluates drugs in terms of their benefit-risk profile when used in older patients. The aim is to reduce the number of potentially inadequate medications in patients aged over 65 years old [36]. In an emergency setting, the tests to record the acute status were carried out directly preoperatively. Tests such as sleep quality and well-being, which cover a longer period before the operation, were carried out postoperatively if not otherwise possible. Through this, the study gains a broad spectrum of possible risk factors while using validated and clinically approved assessment tools.

**Depiction of the pre- and postoperative parameters.**

**Preoperative parameters**

- Age (years), gender, and BMI
- American Society of Anesthesiologists classification
- Comorbidities, risk factors, previous radio and chemotherapy
- Preoperative medication and calculation of anticholinergic load (anticholinergic burden score)
- Delirium Risk Assessment Tool
- Routine laboratory values

**Geriatric assessment**

- Katz index
- Instrumental activities of daily living score according to Lawton and Brody
- Mobility and strength
- de Morton Mobility Index
- Handgrip strength (Hydraulic hand dynamometer)
- Sit-to-stand test
- Emotion World Health Organization-5 scale
- Nutritional status
- Mini Nutritional Assessment Short-Form
- Skinfold thickness over the triceps muscle
- Brain performance
- 6-Item-Screener
- Clock-Drawing-Test
- Hearing ability
- Hearing Handicap Inventory for the Elderly-Short Form
- Sleep disorders or sleep quality
  Pittsburgh Sleep Quality Index
  Insomnia Severity Index
  STOP-BANG–Risk score for obstructive sleep apneas
- Pain assessment

**Postoperative parameters**

- Nursing Delirium Screening Scale (routinely performed in the recovery room by anesthesia)
- Confusion Assessment Method
- Confusion Assessment Method-Intensive Care Unit
- Alertness, Abbreviated Mental Test 4, Attention, Acute Chance or Fluctuating Course
- postoperative pain assessment
- Mobility (de Morton Mobility Index, instrumental activities of daily living, and Katz index) before discharge

**Follow-up**

- Instrumental activities of daily living
- Katz index
- G8 screening test
- Pain assessment
- World Health Organization-5 scale

Testing is done every 8 hours in the Intensive Care Unit Confusion Assessment Method-Intensive Care Unit and twice daily in the normal ward (Confusion Assessment Method and Alertness, Abbreviated Mental Test 4) until postoperative day 7 or discharge [37]. Further testing is done as needed or after revision, operation, and so on.

**Figure 2:**
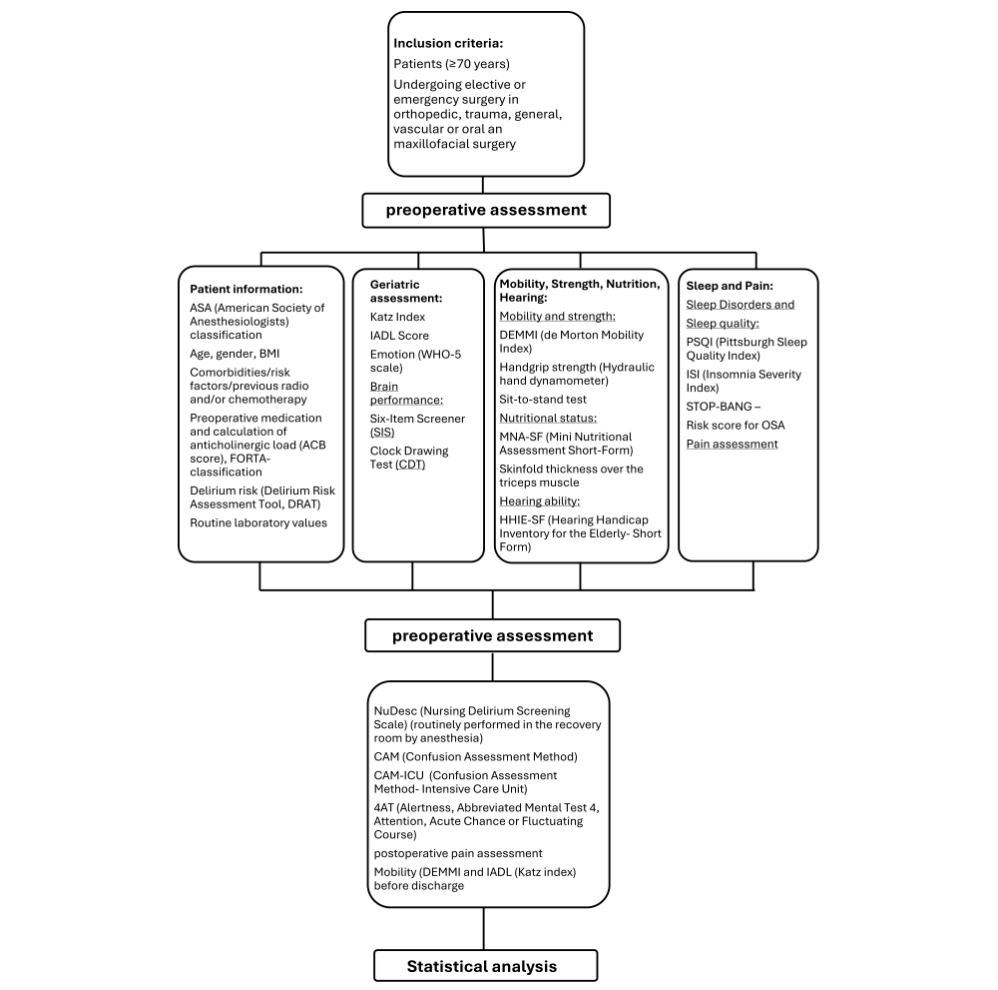
Overview of preoperative evaluation, pre- and postoperative assessment, and analysis. 4AT: Alertness, Abbreviated Mental Test 4, Attention, Acute Chance or Fluctuating Course; ACB: anticholinergic burden; ADL: activities of daily living; ASA: American Society of Anesthesiologists; CAM: Confusion Assessment Method; CAM-ICU: Confusion Assessment Method-Intensive Care Unit; DEMMI: de Morton Mobility Index; DRAT: Delirium Risk Assessment Tool; FORTA: Fit For The Aged; HHIE-SF: Hearing Handicap Inventory for the Elderly-Short Form; IADL: instrumental activities of daily living; ISI: Insomnia Severity Index; MNA-SF: Mini Nutritional Assessment Short-Form; Nu-Desc: Nursing Delirium Screening Scale; OSA: obstructive sleep apnea; PSQI: Pittsburgh Sleep Quality Index; WHO: World Health Organization.

Patients are assessed perioperatively during the daily ward rounds with a focus on their general condition and the presence of delirium. The instruments NuDesc, Confusion Assessment Method, which are routinely recorded in-house, and the Alertness, Abbreviated Mental Test 4, which was introduced in 2020, are used for this purpose [38]. The simultaneous use of these instruments also allows their comparison regarding their quality criteria and may lead to a reduction in monitoring effort in the medium term if one of these instruments proves to be superior (Figure 2).

#### Follow-Up

We plan to contact patients by telephone 3, 6, and 12 months after discharge to inquire about their current health status. Here the patients are asked about changes in sleeping behavior, changes in medication, changes in mobility, and possible rehabilitation treatment. Furthermore, ADL and Katz, G8 Screening Test, a pain assessment, and the World Health Organization–5 scale are recorded. Bellera et al [39] created the G8 geriatric screening test, which evaluates drug use, general health, age, mobility, nutritional state, and cognitive status.

#### Evaluation Outcomes

We aim to determine the frequency of perioperative delirium in patients after surgical treatment in the Clinic for Orthopedics and Trauma Surgery, in the Clinic for Vascular and Endovascular Surgery, and in the Clinic for Oral and Maxillofacial Facial Plastic Surgery at the University Hospital Düsseldorf in the intensive and normal care unit. We want to use the comprehensive assessment to create a risk profile for patients with delirium. The individual risk factors are to be evaluated with regard to their use in the context of a specialized geriatric assessment.

### Statistical Analysis

The evaluation is performed as descriptive statistics with the listing of the frequencies of geriatric problems and perioperative delirium. To investigate the correlation between perioperative delirium and clinical and geriatric parameters, the correlation is determined. The parameters that correlate significantly with the incidence of delirium are examined in a further step in the context of logistic regression analysis for their concrete and independent influence on delirium incidence. Calculations are performed using the statistical program SPSS (version 28.0; IBM Corp) and Jamovi [40]. A *P* value of <.05 is considered significant. If the normal distribution of the dependent variables was confirmed by the Shapiro-Wilk test, homoscedasticity was confirmed by the Levene test and significant outliers were eliminated by boxplots, the mean differences were analyzed using the independent *t* test. The Mann-Whitney *U* test is used to analyze the mean differences of the dependent variables that were not regularly distributed. Chi-square tests are used to analyze bivariate correlations between relevant variables and the occurrence of delirium (delirium vs no delirium). The odds ratios of the relationships are calculated with 95% CIs. Two-sided statistical tests are used, and the α level of .05 is used to determine significance. Significance is defined as a *P* value of less than .05, very significant as a value of less than .01, and highly significant as a value of less than .001. To reduce the possibility of a type I error in repeated testing, Bonferroni correction is used [41]. The factors that cause delirium and that are classified as statistically significant at a significance level of *P*=.05 are determined using binomial logistic regression analysis.

### Ethical Considerations

The study was approved by the Ethics Committee of the Medical Faculty of the Heinrich Heine University Düsseldorf (2022-1810). This study is registered in a publicly accessible database according to DvH2013, § 35 (DRKS-ID; DRKS00028614 [42]). Meeting the requirements for data protection and confidentiality, the data are pseudonymized. It is not possible to identify individual participants or users in figures and illustrations of the manuscript or supplementary materials. No compensation is provided to participants for research.

This study was approved by the ethics review board of the University Hospital of Düsseldorf (2022-1810_2) and all the participants provided written informed consent.

## Results

The comprehensive preoperative geriatric assessment was developed to capture as many potential risk factors for delirium as possible. The findings aim to assist in the development of a specialist and condensed preoperative assessment. Recruitment started in August 2022, with 421 patients already recruited at the time of submission. Initial analyses of the data are to be published at the end of 2024 or the beginning of 2025. Recruitment will continue until August 2025. A final analysis will be issued in a subsequent publication.

## Discussion

We intend to determine the frequency of delirium in the aforementioned specialist disciplines. Furthermore, a specialist risk profile is to be created using a comprehensive geriatric assessment in order to specifically identify patients at risk in the future.

### Relevance of This Study

Delirium occurs particularly in older patients where (1) patients aged 65 years account for up to 40% of all surgical procedures, (2) 50% undergo emergency surgeries, and (3) 75% are affected by surgical mortality [43]. Also, prevalence is particularly increased in those patients who are older, neurocognitively impaired, or emergency-associated patients [44,45]. The reason why delirium is gaining increasing importance in the age group of older patients becomes clear against the background of 2 facts: first, because the total population is getting older, and second, because this group of patients is being operated more and more frequently [46].

Despite the clinical relevance of delirium, there is still no established screening that considers the risk factors of the older patient group in particular so far. This deficiency was the decisive criterion for the establishment of a screening system in the current study, which focuses on the special needs of these older patients. Building on this, the current screening system is therefore intended to create further prevention options in order to meet the needs of the older patient group.

Further on, the need for a new screening system arises from the fact that the presence of delirium is not only a patient-specific problem: postoperative delirium places an additional burden on hospital care in that the average hospital stay is prolonged by an average of 48-72 hours due to postoperative delirium. It is also associated with a 30-day mortality rate of 7%-10% [9,10,47]. The prolonged hospital stays that result, combined with the need for more intensive therapy, lead to increasing costs for the hospital and ultimately for the entire population [48]. A sound assessment that can act preventively on emerging problems and thus prevent the development of delirium, if necessary, would be of both individual and economic benefit.

### Limitations

As described above, predisposing discipline-specific risk factors for the development of postoperative delirium have not yet been thoroughly investigated. The current screening system is intended to circumvent precisely this deficiency and to identify potential risk factors to prevent the development of such delirium in older patients.

Focus was placed on the extraction of clinically relevant examination procedures for the diagnosis of delirium and the elimination of irrelevant procedures, as there is often insufficient time in daily clinical practice to perform comprehensive examinations. General assessments that address the classification of postoperative delirium have already found their way into clinical practice [13,49,50]. However, these do not place a separate focus on the existing risk factors of the group of older patients, who should be given a separate status against the background of the problems described above. What has so far complicated the breakdown of such risk factors is the fact that it is insufficiently possible to distinguish between the normal aging process, the associated increasing physical degradation, and existing medical diseases that accelerate such a decay.

### Method Criticism

The screening system presented here is intended to identify risk factors that can be used for regular screening of older patients. The findings of this study assist in preventing postoperative delirium in the future and reduce the frequency of perioperative problems and mortality in these patients [51,52].

The potential risk factors for the development of postoperative delirium already described above had an influence on the structuring of the screening system presented here. Therefore, the following examination parameters have also found their way into the present screening, which are (1) preoperative history, (2) geriatric assessment, (3) mobility and strength, (4) emotion, (5) nutritional status, (6) brain performance, (7) hearing ability, (8) sleep disorders and sleep quality, (9) pain symptomatology, and (10) postoperative evaluations. They are intended to help extract and further eliminate several different potentially delirium-inducing elements.

### Further Questions

Recording systems have not yet been significantly established, that would be needed for determining dementia, the need for assistance in everyday life, or multimorbidity. These would also be important for determining geriatric care needs.

It has been shown that a geriatric assessment could make perioperative risks more understandable, also in the case of delirium [53]. Marcantonio et al [54] have already elaborated that such a geriatric assessment would reduce delirium by over 30% and severe delirium by over 50%. In this context, the question arises which parameters of such a geriatric screening system could be related to the reduction of delirium frequency.

### Conclusions

We want to investigate whether the risk factors addressed in the assessment are associated with an increase in the delirium rate. The aim is then to reduce this comprehensive assessment to the central aspects to be able to conduct targeted and efficient risk screening.

Many of the known risk factors cannot be easily translated into risk prevention strategies comprehensively in everyday clinical practice. Since each discipline poses different challenges to patients, it is necessary to develop assessments that can be implemented in the individual disciplines.

## Abbreviations

ADL: activities of daily living
DSM-5: Diagnostic and Statistical Manual of Mental Disorders (Fifth Edition)

## Data Availability

The datasets used and analyzed during the current study are available from the corresponding author on reasonable request.
